# Antibiotic pharmacokinetics in infected pleural effusions

**DOI:** 10.1136/thorax-2023-220402

**Published:** 2024-07-04

**Authors:** David T Arnold, Liam Read, Oliver Waddington, Fergus W Hamilton, Sonia Patole, Jessica Hughes, Alice Milne, Alan Noel, Mark Bayliss, Nicholas A Maskell, Alasdair MacGowan

**Affiliations:** 1 Academic Respiratory Unit, University of Bristol, Bristol, UK; 2 Antimicrobial Reference Laboratory, North Bristol NHS Trust, Westbury on Trym, Bristol, UK; 3 Department of Infection Science, North Bristol NHS Trust, Bristol, Bristol, UK; 4 Population Health Sciences, University of Bristol, Bristol, UK

**Keywords:** Pleural Disease, Bacterial Infection, Pneumonia, Respiratory Infection

## Abstract

Pleural infection is usually treated with empirical broad-spectrum antibiotics, but limited data exist on their penetrance into the infected pleural space. We performed a pharmacokinetic study analysing the concentration of five intravenous antibiotics across 146 separate time points in 35 patients (amoxicillin, metronidazole, piperacillin-tazobactam, clindamycin and cotrimoxazole). All antibiotics tested, apart from co-trimoxazole, reach pleural fluid levels equivalent to levels within the blood and well above the relevant minimum inhibitory concentrations. The results demonstrate that concerns about the penetration of commonly used antibiotics, apart from co-trimoxazole, into the infected pleural space are unfounded.

## Introduction

Pleural infection is a serious clinical condition with a mortality of up to 15%.^1^ Patients spend extended periods in the hospital for pleural fluid drainage and intravenous antibiotic administration.^1 2^ There has been concern around the ability of antibiotics to reach therapeutic levels at the site of infection.^2^

## Methods

We recruited patients presenting to a single UK tertiary pleural centre with pleural infection. Patients were consented for regular pleural fluid sampling (via chest tube) with synchronous blood sampling timed with antibiotic administration (1, 2, 4 and 6 hours post dose for a 6-hourly (four times a day) regimen; 1, 4 and 8 hours for an 8-hourly (three times a day) regimen; 1, 4, 8 and 12-hourly (two times a day) for a 12-hourly regimen). The study received ethical approval from the East of Scotland Research Ethics Service (REC reference: 19/ES/0075).

Analytes were assayed using liquid chromatography mass spectrometry assays (LC-MS/MS). Deproteinised samples were injected into a Shimadzu LC system, and gradient separation was performed on a 50×2.1 mm ID 2.6 µm Kinetex XBC18 column using mobile phases containing water/acetonitrile/formic acid (0.1%,v/v). Analytes were selectively detected using a positive ion electrospray source on AB Sciex 4000 QTrap MS. Concentrations in test samples were calculated by Sciex Analyst software using calibrators prepared in the same biological matrix.

Total drug concentrations were plotted with the area under the curve (AUC) from time zero to the next dose determined by the trapezoidal rule. The penetration ratio (PR) for each patient was obtained by dividing the AUC for pleural fluid by the AUC for the blood. Pearson’s correlation coefficient was used to assess the correlation between antibiotic concentrations and pleural fluid pH. A paired t-test was used to compare concentrations between loculated and non-loculated effusions.

## Results

**Figure 1:**
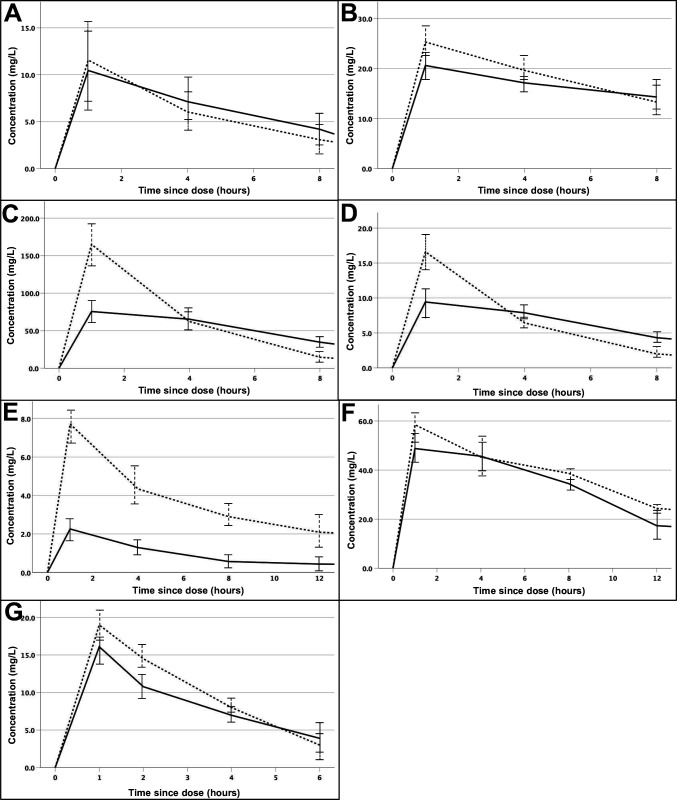
Mean (±SD) intravenous antibiotic concentration-time curves in the blood (dotted line) and pleural fluid (solid line): (A) amoxicillin, (B) metronidazole, (C) piperacillin (piperacillin-tazobactam), (D) tazobactam (piperacillin-tazobactam), (E) trimethoprim (co-trimoxazole), (F) sulphamethoxazole (co-trimoxazole) and (G) clindamycin. Minimum inhibitory concentrations (MICs) (EUCAST) of amoxicillin: *Staphylococcus aureus* 1 mg/L, *Haemophilus influenza* 2 mg/L, *Streptococcus* Milleri group 0.125 mg/L and *Streptococcus pneumoniae* 0.06 mg/L. MICs (EUCAST) of metronidazole: prevotella 1 mg/L, Gram-positive anaerobes (class) 0.25 mg/L and fusobacterium 0.125 mg/L. MICs (EUCAST) of piperacillin-tazobactam: *S. aureus* 2 mg/L, *H. influenza* 0.5 mg/L, *Streptococcus* Milleri group 1 mg/L, *Escherichia coli* 8 mg/L and *S. pneumoniae* 0.064 mg/L. MICs (EUCAST) of trimethoprim-sulfamethoxazole: *S. aureus* 0.25 mg/L, *H. influenza* 0.5 mg/L, *S. pneumoniae* 1 mg/L, *E. coli* 0.5 mg/L. MICs (EUCAST) of clindamycin: *S. aureus* 0.25 mg/L, *Streptococcus* Milleri group 0.25 mg/L and *S. pneumoniae* 0.25 mg/L. The MICs listed for common causative organisms are derived from the EUCAST website (https://www.eucast.org/mic_distributions_and_ecoffs/), which describes the MIC of ‘wild-type’ and, therefore, fully sensitive bacteria.

Thirty-five patients were recruited between October 2019 and March 2022. Six patients met the criteria for frank empyema with the others meeting at least one criterion for pleural infection.^3^ There was a male predominance (77%) with a median age of 76 (IQR 65–79). Mean values (and SD) from baseline pleural fluid analysis were pH of 7.07 (0.22), protein of 39 g/L (12 g/L) and lactate dehydrogenase of 2346 IU (3543 IU). Pleural fluid culture was positive in 7 patients (20%). Full pharmacokinetic analysis could be performed for the antibiotics shown in table 1 with summary curves shown in figure 1.

**Table 1:** Antibiotics assayed, dosing schedule and number of time points assessed

Antibiotic	Route	Dose (frequency)	Timepoints	Patients
Amoxicillin	IV	1000 mg (8 hour)	44	10
Metronidazole	IV	500 mg (8 hour)	31	8
Piperacillin-tazobactam	IV	4500 mg (8 hour)	27	7
Co-trimoxazole	IV	960 mg (12 hours)	24	5
Clindamycin	IV	600 mg (6 hour)	20	5

IV, Intravenous; mg, milligrams.

### Amoxicillin

The AUC_8hr_ for the dosing interval was marginally higher in the pleural fluid compared with the blood (62.8 vs 48.5, PR 1.1) and above the minimum inhibitory concentration (MIC) of causative organisms for the entirety of the dosing schedule.

### Metronidazole

The AUC_8hr_ for metronidazole was similar between the pleural fluid and blood (128.5 vs 151.5, PR 0.84).

### Piperacillin-tazobactam

Piperacillin levels peaked rapidly in the plasma but fell sharply at the 4 hour timepoint.^4^ The AUC_8hr_ was similar between the pleural fluid and blood (465 vs 648, PR 0.72). The AUC_8hr_ for tazobactam was similar between the pleural fluid and blood (55.7 vs 59.5, respectively) with a ratio to piperacillin of 11.6%.

### Co-trimoxazole

The trimethoprim and sulfamethoxazole constituents of co-trimoxazole were measured separately. The AUC_12hr_ was much lower for trimethoprim in the pleural fluid compared with the blood (13.5 vs 47.2), with a PR of 0.29. The ratio for sulfamethoxazole was more equivalent (AUC_12hr_ 465.0 in the blood vs 425.8 in the pleural fluid, PR 0.92).

### Clindamycin

The AUC_6hr_ for clindamycin was similar between the pleural fluid and blood (41.3 vs 51.3, PR 0.81).

### pH and loculation

Across the compounds tested, there was no correlation between the overall pooled mean antibiotic concentrations and the pH of the pleural fluid on sampling. For example, the mean concentration of amoxicillin was 7.9 mg/L (SD 10.2) with a correlation coefficient of −0.145, (p=0.49) (see online supplemental table S1). There was also no mean difference between the blood and pleural fluid antibiotic concentrations depending on the presence and severity of loculations within the effusion (for amoxicillin, metronidazole, piperacillin-tazobactam). For example, the mean difference in amoxicillin concentration was 0.59 mg/L (SD 17.5) in non-loculated effusions versus −1.1 mg/L (SD 11.4) in loculated effusions (p=0.77) (see online supplemental table S2).

10.1136/thorax-2023-220402.supp1 Supplementary data

## Discussion

This is the largest study of antibiotic pharmacokinetics performed in infected pleural effusions. We have demonstrated that commonly used antibiotics such as amoxicillin, metronidazole, piperacillin-tazobactam and clindamycin reach levels within the pleural fluid equivalent to that in the blood and above the MIC for bacteria known to cause pleural infection. The trimethoprim element of co-trimoxazole did not reach the pleural fluid adequately, raising concerns about its use in established or developing pleural infection.

Despite antibiotic recommendations in guidelines and across study protocols, studies on the pleural penetration of many of these antibiotics have not been performed.^5^ The most widely cited evidence on antibiotic concentrations in infected pleural spaces is obtained from rabbit models of turpentine-induced pleural infection.^6^ In 1987, Shohet demonstrated that gentamicin had diminished efficacy in pleural infection, leading to reduced aminoglycoside use for pleural infection.^7^ Given the stark differences in conclusions compared with human pharmacokinetic studies, results from animal models should be interpreted with caution.^2^

Studies of antibiotic pharmacokinetics in human pleural infection are limited in sample size, methodology and antibiotic regimens. A review by Lau *et al* reported that across the relevant five studies, most antibiotics had been assessed following a single dose in one to three patients with pleural infection. Thys *et al* measured aminoglycoside levels after a single intravenous dose in patients with uninfected versus infected purulent pleural infection (n=19 and n=11, respectively). In the infected effusions, levels were either undetectable or significantly lower than plasma, demonstrating the importance of performing pharmacokinetic studies using infected pleural fluid.

This study has demonstrated that the pleural concentrations of amoxicillin, metronidazole, piperacillin-tazobactam and clindamycin are equivalent to that of the blood levels.^6^ Pleural fluid trimethoprim levels were much lower than in the blood across the dosing schedule. There is no previous literature on the pharmacokinetics of trimethoprim within the infected pleural fluid. Extrapolating from other encapsulated infections, there is evidence of poor penetration of co-trimoxazole into the gall bladder during acute cholecystitis.^8^ When compared with the MIC for gram positives that commonly cause pleural infection (eg, *Streptococcus pneumoniae* and the *Streptococcus* virdans group) levels for trimethoprim were inadequate.^9^

We were not able to perform sub-group analysis for individual antibiotics for factors such as the patient’s age, fluid pH or degree of pleural loculation. Second, like many pharmacokinetic studies, comparative MICs have been extrapolated from studies of wild-type bacteria as opposed to those with resistance patterns.

## Conclusion

The commonly used antibiotics such as amoxicillin, metronidazole, piperacillin-tazobactam and clindamycin reached levels equivalent to the blood within infected pleural fluid. Low penetration of trimethoprim into the pleural space raised concerns about the use of cotrimoxazole for patients with pleural infection or parapneumonic effusions.
