# Supplementary material for: Antibiotic pharmacokinetics in infected pleural effusions
Source: Thorax. 2024 Jul 4;79(9):883–5. doi: 10.1136/thorax-2023-220402 (PMC11347206; doi:10.1136/thorax-2023-220402)
Supplement: Supplementary data [file thorax-2023-220402supp001.pdf]

SUPPLEMENTARY TABLES

*TABLE S1: Correlation of pleural antibiotic concentrations and pleural pH**

Antibiotic	pH median (range)	Mean concentration (range)	Correlation coefficient	p-value
Amoxicillin	7.2 (6.8-7.3)	7.9 (1.3-31.0)	-0.145	0.49
Metronidazole	7.15 (6.8-7.2)	17.8 (7.8-26.0)	0.163	0.47
Piperacillin	7.2 (6.9-7.37)	43.1 (2.4-111.0)	-0.211	0.42
Tazobactam	7.2 (6.9-7.37)	5.5 (0.4-17.9)	0.079	0.76

*Across the 4 compounds with the most data available, namely amoxicillin, metronidazole, piperacillin

TABLE S2: Mean differences with standard deviation between plasma and pleural fluid antibiotic concentrations depending on degree of pleural loculation on ultrasound.

Degree of loculation	Non-loculated	Mild	Moderate	Heavy	p-value *
Example image	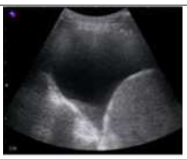	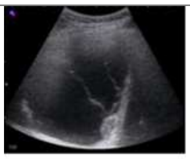	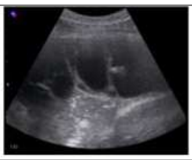	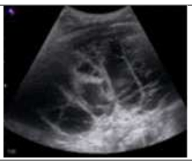	
Amoxicillin	0.59 (17.5)	0.69 (9.34)	-2.1 (10.1)	-4.1 (15.3)	0.77
Metronidazole	N/S	N/S	3.3 (7.7)	2.2 (2.8)	N/P
Piperacillin	20.6 (50.5)	8.4 (65.9)	45.1 (26.7)	2.8 (28.7)	0.57
Tazobactam	0.36 (5.1)	2.4 (1.5)	0.67 (6.4)	-0.8 (2.8)	0.70

*Non-loculated versus loculated (all degrees), N/S- no sample, N/P- not performed
